# Whole genome sequence and analysis of the Marwari horse breed and its genetic origin

**DOI:** 10.1186/1471-2164-15-S9-S4

**Published:** 2014-12-08

**Authors:** JeHoon Jun, Yun Sung Cho, Haejin Hu, Hak-Min Kim, Sungwoong Jho, Priyvrat Gadhvi, Kyung Mi Park, Jeongheui Lim, Woon Kee Paek, Kyudong Han, Andrea Manica, Jeremy S Edwards, Jong Bhak

**Affiliations:** 1 Personal Genomics Institute, Genome Research Foundation, Suwon 443-270, Republic of Korea; 2 The Genomics Institute, Biomedical Engineering Department, UNIST, Ulsan, Republic of Korea; 3 Theragen BiO Institute, TheragenEtex, Suwon 443-270, Republic of Korea; 4 National Science Museum, Daejeon 305-705, Republic of Korea; 5 Department of Nanobiomedical Science & BK21 PLUS NBM Global Research Center for Regenerative Medicine, Dankook University, Cheonan, 330-714, Republic of Korea; 6 DKU-Theragen institute for NGS analysis (DTiNa), Cheonan, 330-714, Republic of Korea; 7 Evolutionary Ecology Group, Department of Zoology, University of Cambridge, Cambridge, UK; 8 Department of Chemistry and Chemical Biology, Department of Molecular Genetics and Microbiology, Department of Chemical and Nuclear Engineering, Cancer Research and Treatment Center, University of New Mexico, Albuquerque, NM 87106, USA

**Keywords:** Marwari, Horse, *Equus ferus caballus*, Whole-genome sequencing, Genome

## Abstract

**Background:**

The horse (*Equus ferus caballus*) is one of the earliest domesticated species and has played an important role in the development of human societies over the past 5,000 years. In this study, we characterized the genome of the Marwari horse, a rare breed with unique phenotypic characteristics, including inwardly turned ear tips. It is thought to have originated from the crossbreeding of local Indian ponies with Arabian horses beginning in the 12th century.

**Results:**

We generated 101 Gb (~30 × coverage) of whole genome sequences from a Marwari horse using the Illumina HiSeq2000 sequencer. The sequences were mapped to the horse reference genome at a mapping rate of ~98% and with ~95% of the genome having at least 10 × coverage. A total of 5.9 million single nucleotide variations, 0.6 million small insertions or deletions, and 2,569 copy number variation blocks were identified. We confirmed a strong Arabian and Mongolian component in the Marwari genome. Novel variants from the Marwari sequences were annotated, and were found to be enriched in olfactory functions. Additionally, we suggest a potential functional genetic variant in the *TSHZ1 *gene (p.Ala344>Val) associated with the inward-turning ear tip shape of the Marwari horses.

**Conclusions:**

Here, we present an analysis of the Marwari horse genome. This is the first genomic data for an Asian breed, and is an invaluable resource for future studies of genetic variation associated with phenotypes and diseases in horses.

## Background

The horse (*Equus ferus caballus*) was one of the earliest domesticated species and has played numerous important roles in human societies: acting as a source of food, a means of transport, for draught and agricultural work, and for sport, hunting, and warfare [1]. Horse domestication is believed to have started in the western Asian steppes approximately 5,500 years ago, and quickly spread across the Eurasian continent, with herds being augmented by the recruitment of local wild horses [2]. Domestication in the Iberian Peninsula might have represented an additional independent episode, involving horses that survived in a steppe refuge following the reforestation of Central Europe during the Holocene [3].

The horse reference genome has provided fundamental genomic information on the equine lineage and has been used for improving the health and performance of horses [1,4]. Horses exhibit 214 genetic traits and/or diseases that are similar to those of humans [5]. To date, several horse whole genomes have been sequenced and analyzed [4,6]. In 2012, the first whole genome re-sequencing analysis was conducted on the Quarter Horse breed to identify novel genetic variants [4]. In 2013, divergence times among horse fossils, donkey, Przewalski's horse, and several domestic horses were estimated, together with their demographic history [6]. However, currently available whole genome sequences of modern horses only comprise western Eurasian breeds.

Over the centuries, more than 400 distinct horse breeds have been established by genetic selection for a wide number of desired phenotypic traits [7]. The Marwari (also known as Malani) horse is a rare breed from the Marwar region of India, and is one of six distinct horse breeds of India. They are believed to be descended from native Indian ponies, which were crossed with Arabian horses beginning around the 12^th ^century, possibly with some Mongolian influence [8-10]. The Marwari horses were trained to perform complex prancing and leaping movements for ceremonial purposes [11,12]. The Marwari population in India deteriorated in the early 1900s due to improper management of the breeding stock, and only a few thousand purebred Marwari horses remain [12].

Here, we report the first whole genome sequence of a male Marwari horse as one of the Asian breeds and characterize its genetic variations, including single nucleotide variations (SNVs), small insertions/deletions (indels), and copy number variations (CNVs). To investigate relationships among different horse breeds, we carried out a genome-wide comparative analysis using previously reported whole genome sequences of six western Eurasian breeds [4,6], and single nucleotide polymorphism (SNP) array data of 729 horses from 32 worldwide breeds [13]. Our results provide insights into its genetic background and origin, and identify genotypes associated with the Marwari-specific phenotypes.

## Results and discussion

### Whole genome sequencing and variation detection

Genomic DNA was obtained from a blood sample of a male Marwari horse (17 years old) and was sequenced using an Illumina HiSeq2000 sequencer. A total of 112 Gb of paired-end sequence data were produced with a read length of 100 bp and insert sizes of 456 and 462 bp from two genomic libraries (Additional file 2: Figure S1, Figure S2). A total of 1,013,642,417 reads remained after filtering, and 993,802,097 reads were mapped to the horse reference genome (EquCab2.0 from the Ensembl database) with a mapping rate of 98.04%. (Additional file 2: Figure S3, Figure S4). A total of 133,091,136 reads were identified as duplicates and were removed from further analyses (Additional file 1: Table S1). To enhance the mapping quality, we applied the IndelRealigner algorithm to the de-duplicated reads. A total of 44,835,563 (5.2%) reads were realigned, and the average mapping quality increased from 53.11 to 53.16 (from 29.33 to 43.32 in the realigned reads). The whole genome sequences covered 95.6% of the reference genome at 10 × or greater depth.

To identify novel genomic sequences, we performed a *de novo *assembly using the unmapped reads (1.8 Gb) to the horse reference genome. A total of 120,159 contigs (24,781,670 bases in length and 227 bp of contig N50 size) were assembled. After mapping the contigs to the reference genome, we found that 25,614 contigs (4,855,119 bases in length and 196 bp of contig N50 size) did not match the reference sequences; indicating that they may be novel regions specific to the Marwari horse breed (Additional file 1: Table S2). To identify the biological functions of these novel regions, the un-matched contigs were further analyzed by BLAST searches using the NCBI protein database. However, none of the contigs significantly matched the known protein database (Additional file 2: Figure S5).

Comparing the Marwari sequence to the reference genome, approximately 5.9 million SNVs and 0.6 million indels were identified (Table 1). Estimates of SNP rate and heterozygosity of the Marwari were similar to those of other horse breeds (Arabian, Icelandic, Norwegian Fjord, Quarter, Standardbred, and Thoroughbred) (Additional file 1: Table S3). We assessed the mutational frequency at the single nucleotide level in the Marwari and compared it to estimates from other breeds (Additional file 1 Table S4). Interestingly, we found that the prevalent mutation types were not consistent among horse breeds. The mutation spectrum of the Marwari was dominated by C>T (G>A) transitions; a pattern which was also observed in the Icelandic, Norwegian Fjord, and Quarter horses. Conversely, the genomes of the Arabian, Standardbred, and Thoroughbred horses were dominated by A>G (T>C) transitions. A significant association between the mutation spectrum and horse breed (p-value < 0.001) was found when we applied a chi-square test using SPSS [14] to statistically compare the differences in the mutation spectrums among the breeds.

**Table 1:** Variants in the Marwari horse genome.

Description	SNVs			Indels		
	**Homozygous**	**Heterozygous**	**Novel**	**Homozygous**	**Heterozygous**	**Novel**

Total	2,383,702	3,539,864	1,577,725	343,789	234,266	249,609
INTERGENIC	1,565,078	2,352,370	1,060,195	215,679	153,412	164,564
INTRAGENIC	3,474	5,134	1,919	556	332	329
UPSTREAM	113,184	168,184	74,923	18,300	10,582	11,178
DOWNSTREAM	111,918	166,365	75,592	17,866	11,504	12,203
UTR_5_PRIME	600	684	279	171	27	25
UTR_3_PRIME	1,188	1,660	802	264	129	149
INTRON	569,725	817,541	351,960	89,394	57,856	60,614
Noncoding exon variant	3,259	4,368	3,433	280	198	244
Synonymous mutation	8,053	12,586	4,209	0	0	0
Nonsynonymous mutation	7,223	10,972	4,413	0	0	0
Indels in coding region	0	0	0	1,279	226	303

The Marwari genome consisted of 2,383,702 (40.2%) homozygous and 3,539,864 (59.8%) heterozygous SNVs (Table 1). Among them, 18,195 were found to be nonsynonymous SNVs (nsSNVs). When the Marwari variants were compared to those previously reported from the genomes of other breeds [4,6] and the horse SNP database from the Broad Institute, 1,577,725 SNVs and 249,609 indels were novel variants. Of these, 4,716 variants (4,413 nsSNVs and 303 indels in coding regions) represented amino acid changes which were found in 2,770 genes (2,584 genes with nsSNVs, 279 genes with indels in coding regions, and 93 genes with nsSNVs and indels in coding regions simultaneously). To annotate the variants using well-known functional databases, human orthologs were retrieved from the Ensembl BioMart utility. A total of 1,970 of the 2,770 genes had human orthologs, and 1,896 genes were annotated using the DAVID Bioinformatics Resource 6.7 [15]. The genes with nsSNVs and/or indels in coding regions were highly enriched in olfactory functions (Additional file 1: Tables S5 and S6).

Copy number variations (CNVs) were identified using the R library "ReadDepth package" [16]. A total of 2,579 CNVs, including 869 gain and 1,710 loss blocks, were identified in the Marwari genome. The sizes ranged from 3 Kb to 6.43 Mb with an average length of 56 Kb. The CNV region (140 Mb in length) contained 2,504 genes which were duplicated (1,138 genes) or deleted (1,366 genes) (Additional file 1: Table S7). From the functional enrichment analysis, we found that the duplicated genes were enriched in olfactory function, whereas the deleted genes were enriched in immune regulation and metabolic processes (Additional file 1:Table S8, Table S9, Table S10, and Table S11).

### Relatedness to other horse breeds

We constructed a phylogenetic tree using SNVs found in the whole genome data of the seven horse breeds (Arabian, Icelandic, Marwari, Norwegian Fjord, Quarter, Standardbred, and Thoroughbred) [4,6]. We identified 11,377,736 nucleotide positions that were commonly found in the seven horse genomes. A total of 25,854 nucleotide positions were used for phylogenetic analysis after filtering for minor allele frequency (MAF), genotyping rate, and linkage disequilibrium (LD). We found that the Marwari horse is most closely related to the Arabian breed (Additional file 2: Figure S6), while the Icelandic horse and Norwegian Fjord were the most distinct from the other breeds, all of which are known to descend from Arabian horses [17,18].

**Figure 1:**
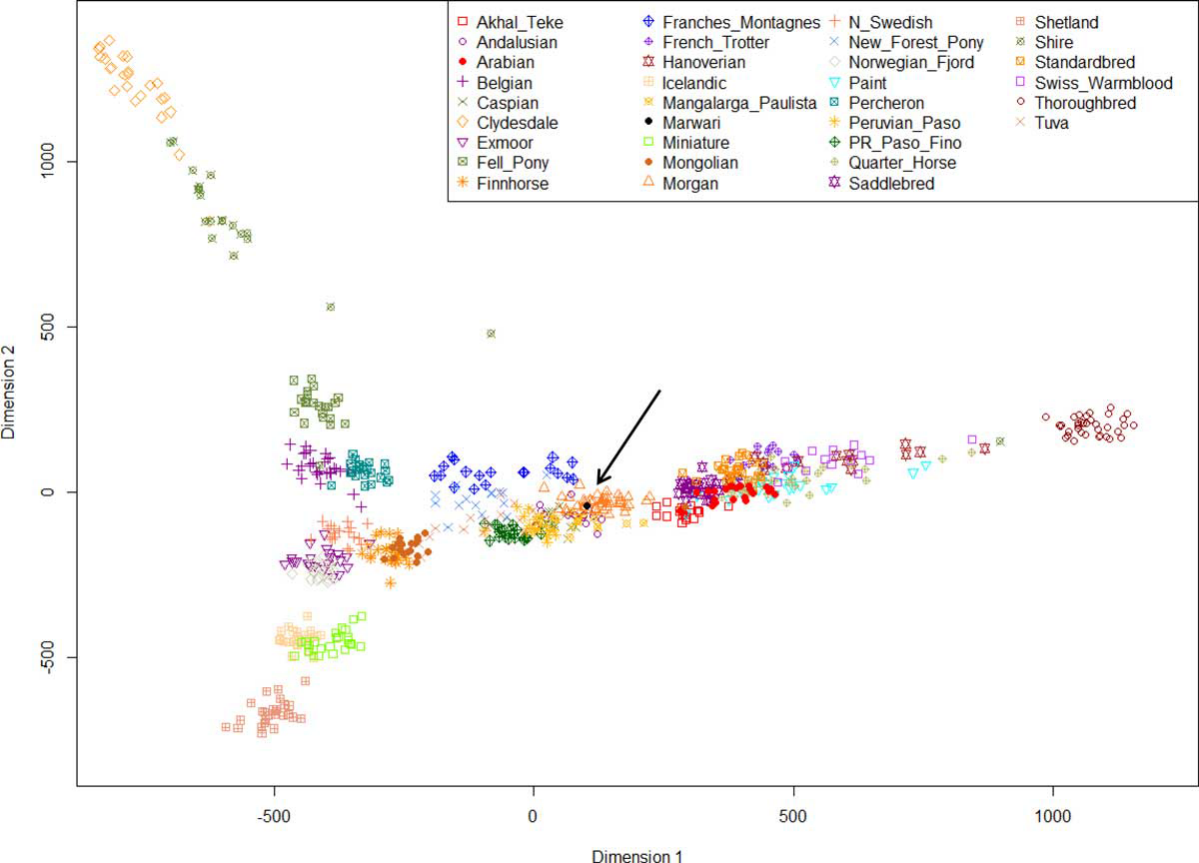
**Multidimensional scaling plot derived from a Marwari horse and other horse breeds**. Black arrow indicates the Marwari horse.

To further explore the relationships among breeds, we compared the Marwari horse genome data with SNP array data from 729 individual horses belonging to 32 domestic breeds [13]. A total of 54,330 nucleotide positions were shared across all individuals including the Marwari horse. After pruning as described above, 10,554 nucleotide positions were used for the comparative analyses. We calculated pairwise genetic distances and conducted multidimensional scaling (MDS) to visualize the relationships among the horse breeds (Figure 1). The Marwari horse fell together with Iberian-lineage breeds, such as the Andalusian, Mangalarga Paulista, Peruvian Paso, and Morgan horse breeds, all of which are known to have an Arabian ancestry [19-22]. Additionally, we found that the Marwari horse fell between Arabian and Mongolian horses, indicating their dual genetic influences on the Marwari horse as previously suggested [8-10].

**Figure 2:**
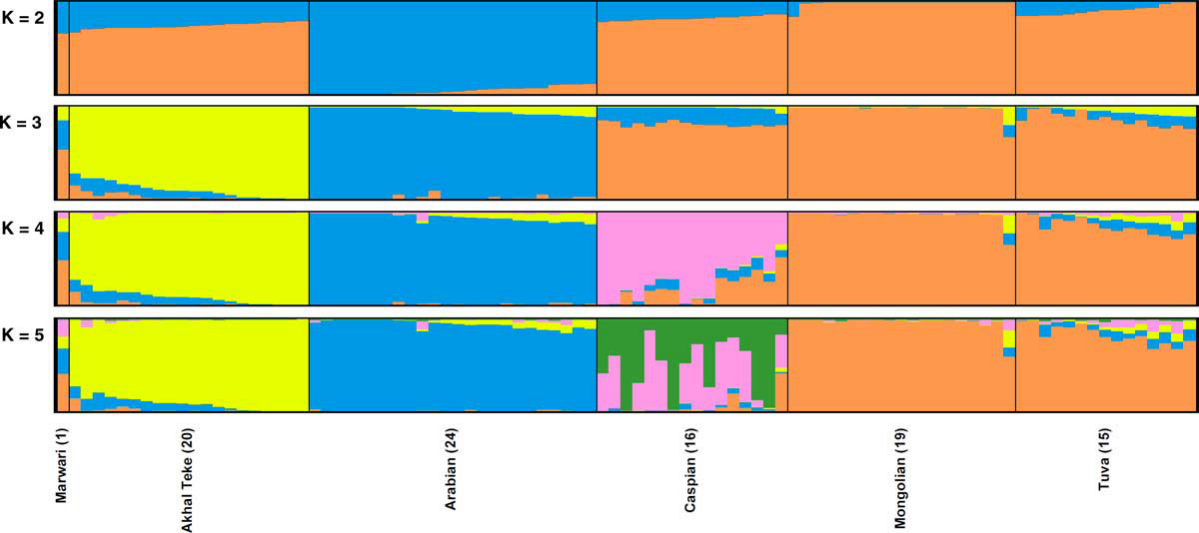
**STRUCTURE analysis using Marwari and Asian horse breeds**. For all K values, the Marwari has genetic affinities to both Arabian (blue) and Mongolian horses (orange).

We applied the STRUCTURE program [23,24] to estimate the genetic composition of the Asian horse breeds including the Marwari horse. For K = 2 groups, the Arabian horses were strongly separated from Mongolian horses, and the genetic composition of the Marwari horse was composed of alleles clustering with both the Mongolian horse (65.8%) and the Arabian horse (34.2%) (Figure 2). Other Asian breeds (Akhal Teke, Caspian, and Tuva) also showed genetic admixture between Arabian and Mongolian horses. From K = 3 to K = 5, the Marwari had high genetic components of both Arabian and Mongolian horses, whereas Akhal Teke and Caspian horses were mostly assigned to other clusters. These results indicate that the Marwari is genetically closely related to the Arabian and Mongolian horses. It is unclear whether the latter relationship represents direct genetic input from Mongolian horses or whether these horses are the closest population to the Indian ponies from which the Marwari is thought to have descended [8-10]. Further analysis including Indian ponies and Marwari horses will be required to distinguish the relative importance of these two scenarios, which are not mutually exclusive.

### Phenotype association of the identified variants

**Figure 3:**
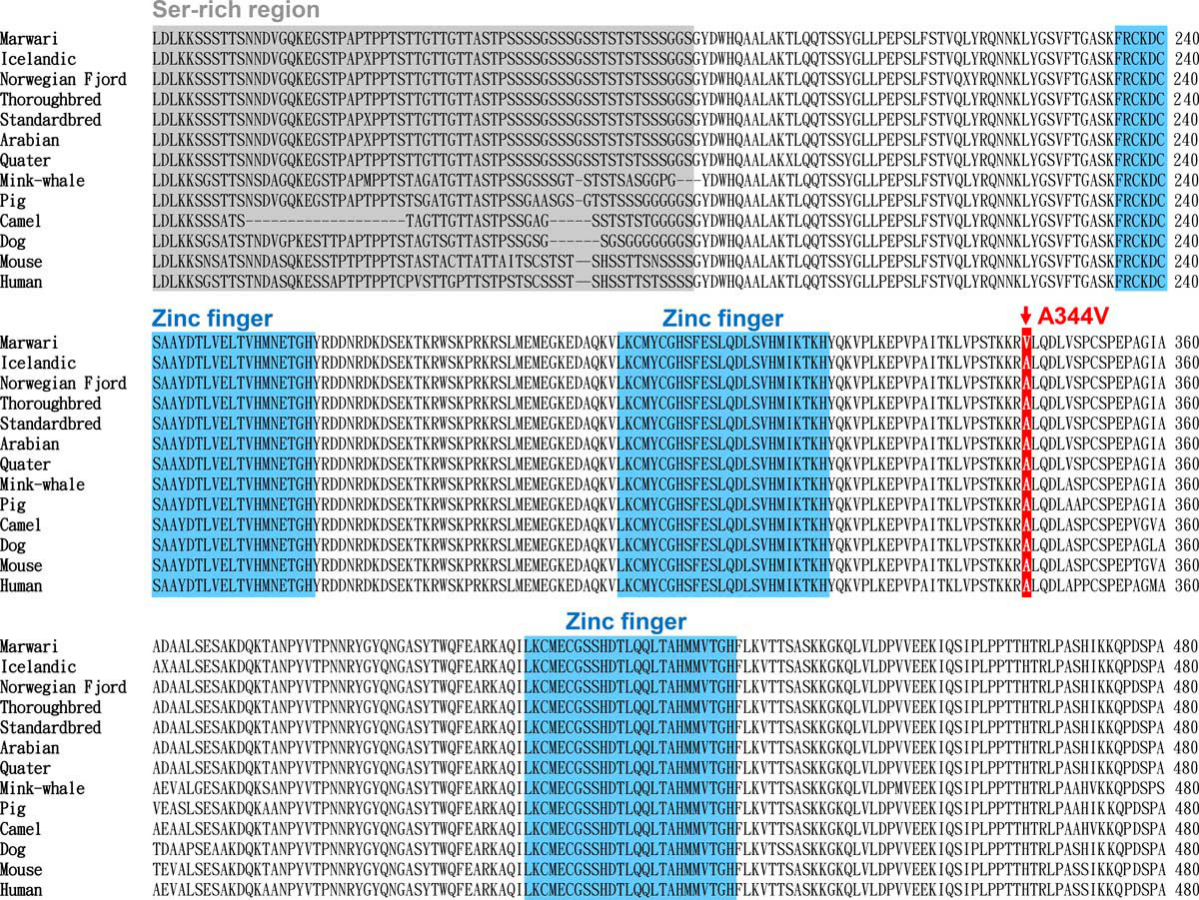
**Partial alignment of TSHZ1 amino acid sequences among horse breeds and vertebrate species**. Red rectangle indicates a Marwari horse-specific amino acid change (A344V). Gray and blue rectangles indicate a Ser-rich region and Zinc fingers, respectively.

To provide insight into the unique Marwari phenotypes, we investigated amino acid changes specific to this breed compared to those of other breeds (Arabian, Icelandic, Norwegian Fjord, Quarter, Standardbred, and Thoroughbred). A total of 343 amino acid changes in 236 genes were unique to the Marwari horse. Among the 236 genes, 75 genes included one or more amino acid changes predicted by the PolyPhen2 program to alter protein function [25] (Additional file 1: Table S12). Interestingly, the teashirt zinc finger family member 1 (*TSHZ1*) gene had a homozygous p.Ala344>Val variant (Figure 3). *TSHZ1 *is involved in transcriptional regulation of developmental processes and is associated with congenital aural atresia in humans, a malformation of the ear occurring in approximately 1 in 10,000 births [26,27]. Additionally, *TSHZ1*-deficient mice show malformations in the middle ear components [28]. Therefore, the A334V amino acid change in *TSHZ1 *is a strong candidate as the genetic factor responsible for the inward-turning ear tips characteristic of the Marwari breed. A future genomic comparison with the Kathiawari horse, which also has inward-turning ear tips, might support to this prediction.

After annotating the Marwari variants for their known disease and trait information [26-55] (Table 2), we found that this breed has a homozygous variant for the g.27991841A>G mutation in the *SCL26A2 *gene, which causes autosomal recessive chondrodysplasia in equine. Other variants were associated with racing endurance in Thoroughbred horses (g.32772871C>T in *COX4I1*, g.40279726C>T in *ACN9*), horse size (g.81481065C>T in *HMGA2*, g.23259732G>A in *LASP1*), and pattern of locomotion (g.22999655C>A in *DMRT3*).

**Table 2:** Genetic variants for known traits and diseases.

PMID	CHR	Coordinate	Gene	Phenotype	Associated Genotype	Marwari Genotype
21070277 [29]	1	74,842,283	ACTN2	Racing performance	A>G	A/A
20353955 [30]	1	108,249,293	TRPM1	Leopard complex spotting and congenital stationary night blindness	C>T	C/C
17498917 [31]	1	128,056,148	PPIB	Hereditary equine regional dermal asthenia	G>A	G/G
20419149 [32]	1	138,235,715	MYO5A	Lavender foal syndrome	Del 1 bp	neg
21070277 [29]	3	32,772,871	COX4I1	Racing performance	C>T	**T/C**
8995760 [33]	3	36,259,552	MC1R	Chestnut coat color	C>T	C/C
11086549 [34]	3	36,259,554	MC1R	Chestnut coat color	G>A	G/G
16284805 [35]	3	77,735,520	KIT	Sabino spotting	A>T	A/A
18253033 [36]	3	77,740,163	KIT	Tobiano spotting pattern	G>A	G/G
22808074 [37], 22615965 [38]	3	105,547,002	LCORL, NCAPG	Large body size	T>C	T/T
21070277 [29]	4	40,279,726	ACN9	Racing performance	C>T	**T/T**
12230513 [39]	5	20,256,789	LAMC2	Junctional epidermolysis bullosa	Ins C	neg
17029645 [40]	6	73,665,304	PMEL17	Silver coat color	G>A	G/G
22808074 [37]	6	81,481,065	HMGA2	Large body size	C>T	**T/T**
19016681 [41]	8	45,603,643- 45,610,231	LAMA3	Junctional epidermolysis bullosa	Del 6589	neg
9103416 [42]	9	35,528,429	DNAPK	Severe combined immunodeficiency	Del 5 bp	neg
22615965 [38]	9	74,795,013	ZFAT	Wither height	C>T	C/C
22808074 [37]	9	75,550,059	ZFAT	Large body size	C>T	C/C
15318347 [43]	10	9,554,699	RYR1	Malignant hyperthermia	C>G	C/C
21059062 [44]	10	15,884,567	CKM	Racing performance	G>A	G/G
18358695 [45]	10	18,940,324	GYS1	Polysaccharide storage myopathy	C>T	C/C
7623088 [46]	11	15,500,439	SCN4A	Equine hyperkalemic periodic paralysis	C>T	C/C
22808074 [47]	11	23,259,732	LASP1	Large body size	G>A	**A/A**
21070269 [47]	14	3,761,254	PROP1	Dwarfism	G>C	G/G
18802473 [48]	14	26,701,092	SLC36A1	Champagne dilution	G>C	G/G
17901700 [49]	14	27,991,841	SCL26A2	Autosomal recessively inherited chondrodysplasia	A>G	**G/G**
9580670 [50]	17	50,624,658	EDNRB	Lethal white foal syndrome	GA>CT	GA/GA
20932346 [51]	18	66,493,737	MSTN	Optimum racing performance	T >C	T/T
12605854 [52]	21	30,666,626	SLC45A2	Cream coat color	G>A	G/G
21059062 [44]	22	22,684,390	COX4I2	Racing performance	C>T	C/C
11353392 [53]	22	25,168,567	ASIP	Black and bay color	Del 11 bp	Neg
22932389 [54]	23	22,999,655	DMRT3	Pattern of locomotion (altered gait)	C>A	**A/C**
18641652 [55]	25	6,574,013- 6,581,600	STX17	Gray coat color	Dup 4600	neg

### Selection in the equid lineage

We assessed the signatures of selection in the equid lineage using the *d_N_*/*d_S _*(nonsynonymous substitutions per nonsynonymous site to synonymous substitutions per synonymous site) ratio [56]. A consensus horse (equid) sequence was constructed by integrating all of the available breed genomes (Arabian, Icelandic, Marwari, Norwegian Fjord, Quarter, Standardbred, and Thoroughbred) in an attempt to remove breed specificity and to include an Asian breed component via the central Asian heritage of the Marwari (in contrast to the western Eurasian breeds for which whole genomes had been previously sequenced). A total of 7,711 out of 22,305 genes in the horse reference genome were substituted by the consensus sequences. Using the protein sequences of seven non-horse genomes (camel, pig, cow, minke whale, dog, mouse, and human), 5,459 orthologous gene families were constructed using OrthoMCL [57]. Using alignments of these gene families to estimate *d_N_*/*d_S_*, we identified 188 genes under selection in the horse genome (Additional file 1: Table S13). The selected genes were particularly enriched in immune response (immune effector process, leukocyte mediated immunity, positive regulation of immune system process, and defense response) and possible motor ability (T-tubule, muscle contraction, and regulation of heart contraction) functions (Additional file 1: Table S14). Over evolutionary time, the horse has developed increased speed and its musculature has become specialized for efficient strides [58,59]. It is therefore possible that the motor activity-associated genes we identified to be under positive selection have contributed to the muscular efficiency seen in modern horses.

## Conclusion

Our study provides the first whole genome sequences and analyses of the Marwari, an Asian horse breed. Comparing the Marwari genome to the horse reference genome, approximately 5.9 million SNVs and 0.6 million indels, including 4,716 variants that cause amino acid changes, were identified. We found a clear Arabian and Mongolian component in the Marwari genome, although further work is needed to confirm whether modern Marwari horses also descended from Indian ponies. We analyzed the Marwari variants and found a candidate SNV determining its characteristic inward-turning ear tips. Additionally, we investigated selection in the horse genome through comparisons with other mammalian genomes. By creating a consensus sequence that included information on an Asian breed, we found a number of genetic signatures of selection, providing insights into possible evolutionary and environmental adaptations in the equid lineage. The whole genome sequencing data from the Marwari horse provides a rich and diverse genomic resource that can be used to improve our understanding of animal domestication and will likely be useful in future studies of phenotypes and disease.

## Methods

### Sample preparation and whole genome sequencing

Genomic DNA was extracted from the blood of a 17 year old male Marwari horse with the XcelGene Blood gDNA Mini Kit (Xcelris Labs Ltd, Gujarat, India) following the manufacturer's protocol. Two genomic libraries with insert sizes of 456 and 462 bp were constructed at Theragen BiO Institute (TBI), TheragenEtex, Korea. The genomic DNA was sheared using Covaris S series (Covaris, MS, USA). The sheared DNA was end-repaired, A-tailed, and ligated to paired-end adapters, according to the manufacturer's protocol (Truseq DNA Sample Prep Kit v2, Illumina, San Diego, CA, USA). Adapter-ligated fragments were then size selected on a 2% Agarose gel, and the 520-620 bp band was extracted. Gel extraction and column purification were performed using the MinElute Gel Extraction Kit (Qiagen, CA, USA) following the manufacturer's protocol. The ligated DNA fragments containing adapter sequences were enhanced via PCR using adapter-specific primers. Library quality and concentration were determined using an Agilent 2100 BioAnalyzer (Agilent, CA, USA). The libraries were quantified using a KAPA library quantification kit (Kapa Biosystems, MA, USA) according to Illumina's library quantification protocol. Based on the qPCR quantification, the libraries were normalized to 2 nM and denatured using 0.1 N NaOH. Cluster amplification of denatured templates was performed in flow cells according to the manufacturer's protocol (Illumina, CA, USA). Flow cells were paired-end sequenced (2 × 100 bp) on an Illumina HiSeq2000 using HiSeq Sequencing kits. A base-calling pipeline (Sequencing Control Software, Illumina) was used to process the raw fluorescent images and the called sequences.

### Filtering and mapping processes

Before the mapping step, raw reads were filtered using NGS QC toolkit version 2.3 (cutoff read length for high quality, 70%; cutoff quality score, 20) [60]. After the filtering step, clean reads were mapped to the horse reference genome (Ensembl EquCab2.0, release 72) [1] with BWA version 0.7.5a [61] with minimum seed length (-k 15) and Mark shorter split hits as secondary (-M). We realigned the reads using the GATK [62] IndelRealigner algorithm to enhance the mapping quality, and marked duplicate reads using MarkDuplicates from picard-tools version 1.92 (http://broadinstitute.github.io/picard/).

### *De novo *assembly of unmapped reads

We extracted unmapped sequences from aligned Marwari BAM files. To find Marwari specific genomic regions, we assembled unmapped reads using SOAPdenovo2 [63] with "all" mode and multiple K values (ranged from 23 to 63). A total of 120,159 contigs were obtained, and N50 length was 227bp. To identify whether these contigs are in non-reference regions, we aligned contigs to the horse reference genome. A total of 25,614 contigs were not aligned to the reference genome. The non-reference sequences were further analyzed by BLAST to NCBI protein and DNA sequence databases with the criteria E≤10^-5 ^and identity ≥ 70%.

### Variant detection and annotation

Putative variant calls were made using the SAMtools version 0.1.16 [64] mpileup command. In this step, we used the -E option to minimize the noise resulting from pairwise read alignments, and the -A option to use regardless of insert size constraint and/or orientation within pairs. Variants were called using bcftools and then filtered using vcfutils varFilter (minimal depth of 8, maximal depth of 100, Phred scores of SNP call ≥ 30, and no indel present within a 2 bp window) as previously reported [6]. SnpEff [65] was used to annotate the variants. To find unique variants for the Marwari horse, SNVs and small indels were further compared with the horse SNP database that was identified by the Horse Genome Project (http://www.broadinstitute.org/mammals/horse/snp), and other previously reported horse breed genomes [4,6]. Copy number variants based on the differences in sequencing depths were detected using R library "ReadDepth package" with default options. The ReadDepth calculated the thresholds for copy number gain (2.642) and loss (1.380).

### Phylogenetic tree construction

Genotype data were extracted from a total of 11,377,736 single nucleotide positions, which were shared and sufficiently covered regions (> 8 × depth), in the seven horse whole genome data (Arabian, Icelandic, Marwari, Norwegian Fjord, Quarter, Standardbred, and Thoroughbred) [4,6]. The genotyping data were merged, and then filtered to remove those SNP with a genotyping rate of < 0.05 and allele frequency > 0.2 using PLINK [66]. SNPs that were in linkage disequilibrium (LD) were also removed: the merged files were pruned for r^2 ^< 0.1 in PLINK, considering 100 SNP windows and moving 25 SNPs per set (-indep-pairwise 100 25). After the filtering and pruning process, 25,854 SNPs remained and were used for the phylogenetic analysis. RAxML version 7.28-ALPHA [67] was used to generate the parsimony starting trees, and RAxML-Light version 1.0.9 [68] was used to carry out tree inference with the GTRGAMMA model of nucleotide substitutions. A total of 100 bootstrap trees were generated for each phylogeny. The resulting tree was drawn by MEGA6 [69].

### MDS and population structure analyses

Equine SNP array data of 729 individuals belonging to 32 horse breeds were obtained from a previous report [13]. The Marwari horse data used in this analysis were selected from 54,330 nucleotide positions that were derived from the SNP array data. The SNP array and Marwari data were filtered and pruned to remove SNPs with the same cutoffs described above, except that the MAF option was set to --maf < 0.05. A total of 10,554 single nucleotide positions were used for the following comparative analyses.

The MDS plot was drawn in R [70] using the "MASS" library and "canberra" distance metric. STRUCTURE version 2.3.4 [23,24] was used to cluster Asian breeds based on genetic similarity, investigating K values from 2 to 5. Each run for a given K value consisted of a 15,000 steps burn-in and 35,000 MCMC repetitions. We applied a default admixture model and a default assumption that allele frequencies were correlated. The convergence of STRUCTURE runs was evaluated by the equilibrium of alpha. Individual and population clump files were produced with Structure Harvester [71] and visualized in Distruct1.1 [72].

### Orthologous gene family

Protein sequences of cow (*Bos taurus*), dog (*Canis familiaris*), human (*Homo sapiens*), mouse (*Mus musculus*), and pig (*Sus scrofa*) were downloaded from the Ensembl database version 72. Protein sequences of minke whale (*Balaenoptera acutorostrata*) [73] and camel (*Camelus bactrianus*) [74] were obtained from the original publications. A total of eight species were used to identify orthologous gene clusters with OrthoMCL 2.0.9. Pairwise sequence similarities between all protein sequences were calculated using BLASTP with an e-value cutoff of 1E-05. On the basis of the BLASTP results, OrthoMCL was used to perform a Markov clustering algorithm with inflation value (-I) of 1.5. The OrthoMCL was run with an e-value exponent cutoff of -5 and percent match cutoff of 50%. In total, 5,501 orthologous groups were shared by all eight species. The representative sequences for each gene cluster were selected using the longest horse transcript and the corresponding protein sequences of the other species. BLASTP searches (E-value 1E-5 cutoff) between horse and all the other species were used in this process. Finally, we identified 5,459 1:1:1:1:1:1:1:1 orthologs.

### Molecular evolutionary analysis

The phylogenetic tree was constructed from 5,459 single copy ortholog genes. CODEML in PAML 4.5 [75] was used to estimate the *d_N_*/*d_S _*ratio, where *d_N _*indicates nonsynonymous substitution rate and *d_S _*indicates synonymous substitution rate. The *d_N_*/*d_S _*ratio along the horse branch (free-ratio molel) and *d_N_*/*d_S _*ratio for all branches (one-ratio model) were calculated as the branch model. We also applied the branch-site model to further examine potential positive selection [76]. The LRTs (likelihood ratio tests) were applied to assess statistical significance of the branch-site model. We supposed that positively selected genes are that of having a higher *d_N_*/*d_S _*ratio with the free-ratios model than that with the one-ratio model and having p-value < 0.05 from branch-site model.

### Availability of supporting data

Whole genome sequence data was deposited in the SRA database at NCBI with Biosample accession numbers SAMN02767683. SRA of whole genome sequencing can be accessed via reference numbers SRX535352. The data can also be accessed through BioProject accession number PRJNA246445 for the whole genome sequence data.

## Competing interests

The authors declare that they have no competing interests.

## Authors' contributions

Conceived and designed the experiments: JB and WKP. Performed the experiments: KMP. Analyzed the data: JHJ, SJho, YSC, HJH, and JL. Study design, subject recruitment, and sample preparation: PG and JB. Data interpretation: JHJ, SJho, YSC, HJH, and JL. Wrote the paper: JHJ, YSC, HMK, HJH, JE, KH, AM, PG, and JB.

## Supplementary Material

Additional file 1 Click here for file

Additional file 2 Click here for file

## References

[B1] Wade CM Giulotto E Sigurdsson S Zoli M Gnerre S Imsland F Lear TL Adelson DL Bailey E Bellone RR Blocker H Distl O Edgar RC Garber M Leeb T Mauceli E MacLeod JN Penedo MC Raison JM Sharpe T Vogel J Andersson L Antczak DF Biagi T Binns MM Chowdhary BP Coleman SJ Della Valle G Fryc S Guerin G Genome sequence, comparative analysis, and population genetics of the domestic horse Science 2009 326 865 867 10.1126/science.1178158 19892987 PMC3785132

[B2] Warmuth V Eriksson A Bower MA Barker G Barrett E Hanks BK Li S Lomitashvili D Ochir-Goryaeva M Sizonov GV Soyonov V Manica A Reconstructing the origin and spread of horse domestication in the Eurasian steppe Proc Natl Acad Sci USA 2012 109 8202 8206 10.1073/pnas.1111122109 22566639 PMC3361400

[B3] Warmuth V Eriksson A Bower MA Cañon J Cothran G Distl O Glowatzki-Mullis ML Hunt H Luís C do Mar Oom M Yupanqui IT Ząbek T Manica A European Domestic Horses Originated in Two Holocene Refugia PLoS One 2011 6 e18194 10.1371/journal.pone.0018194 21479181 PMC3068172

[B4] Doan R Cohen ND Sawyer J Ghaffari N Johnson CD Dindot SV Whole-Genome Sequencing and Genetic Variant Analysis of a Quarter Horse Mare BMC Genomics 2012 13 78 10.1186/1471-2164-13-78 22340285 PMC3309927

[B5] Online Mendelian Inheritance in Animals http://omia.angis.org.au/home

[B6] Orlando L Ginolhac A Zhang G Froese D Albrechtsen A Stiller M Schubert M Cappellini E Petersen B Moltke I Johnson PL Fumagalli M Vilstrup JT Raghavan M Korneliussen T Malaspinas AS Vogt J Szklarczyk D Kelstrup CD Vinther J Dolocan A Stenderup J Velazquez AM Cahill J Rasmussen M Wang X Min J Zazula GD Seguin-Orlando A Mortensen C Recalibrating Equus evolution using the genome sequence of an early Middle Pleistocene horse Nature 2013 499 74 81 10.1038/nature12323 23803765

[B7] Hendricks B International Encyclopedia of Horse Breeds 1995 Norman: University of Oklahoma Press

[B8] Elwyn Hartley Edwards The Encyclopedia of the Horse 1994 New York: Dorling Kindersley

[B9] Wendy Doniger The Hindus: An Alternative History 2009 New Delhi: Penguin Books

[B10] Behl R Behl J Gupta N Gupta SC Genetic relationships of five Indian horse breeds using microsatellite markers Animal 2007 4 483 488 22444405 10.1017/S1751731107694178

[B11] Dutson Judith Storey's Illustrated Guide to 96 Horse Breeds of North America 2005 North adams: Storey Publishing

[B12] Gupta AK Chauhan M Tandon SN Sonia Genetic diversity and bottleneck studies in the Marwari horse breed J Genet 2005 84 295 301 10.1007/BF02715799 16385161

[B13] Petersen JL Mickelson JR Rendahl AK Valberg SJ Andersson LS Axelsson J Bailey E Bannasch D Binns MM Borges AS Brama P da Câmara Machado A Capomaccio S Cappelli K Cothran EG Distl O Fox-Clipsham L Graves KT Guérin G Haase B Hasegawa T Hemmann K Hill EW Leeb T Lindgren G Lohi H Lopes MS McGivney BA Mikko S Orr N Genome-wide analysis reveals selection for important traits in domestic horse breeds PLoS Genet 2013 9 e1003211 10.1371/journal.pgen.1003211 23349635 PMC3547851

[B14] IBM Corp 2013 IBM SPSS Statistics for Windows, Version 22.0. NY: IBM

[B15] Huang da W Sherman BT Zheng X Yang J Imamichi T Stephens R Lempicki RA Extracting biological meaning from large gene lists with DAVID Curr Protoc Bioinformatics 2009 Chapter 13:Unit 13.11 10.1002/0471250953.bi1311s27 19728287

[B16] Miller CA Hampton O Coarfa C Milosavljevic A ReadDepth: a parallel R package for detecting copy number alterations from short sequencing reads PLoS One 2011 6 e16327 10.1371/journal.pone.0016327 21305028 PMC3031566

[B17] John F Wall Famous Running Horses: Their Forebears and Descendants 2013 Whitefish: Literary Licensing

[B18] Robert Moorman Denhardt The Quarter Horse Running: America's Oldest Breed 2003 Norman: University of Oklahoma Press

[B19] Llamas This is the Spanish Horse 1999 London: J A Allen & Co Ltd

[B20] Milner Godolphin Arabian: Story of the Matchem Line 1990 London: J. A. Allen

[B21] Breed of Livestock http://www.ansi.okstate.edu/breeds/horses/

[B22] International Museum of the HORSE http://www.imh.org/exhibits/online/breeds-of-the-world

[B23] Falush D Stephens M Pritchard JK Inference of population structure using multilocus genotype data: linked loci and1 correlated allele frequencies Genetics 2003 164 1567 1587 12930761 10.1093/genetics/164.4.1567 PMC1462648

[B24] Pritchard JK Stephens M Donnelly P Inference of population structure using multilocus genotype data Genetics 2000 155 945 959 10835412 10.1093/genetics/155.2.945 PMC1461096

[B25] Adzhubei IA Schmidt S Peshkin L Ramensky VE Gerasimova A Bork P Kondrashov AS Sunyaev SR A method and server for predicting damaging missense mutations Nat Methods 2010 7 248 249 10.1038/nmeth0410-248 20354512 PMC2855889

[B26] ALTMANN F Congenital atresia of the ear in man and animals Ann Otol Rhinol Laryngol 1955 64 824 858 10.1177/000348945506400313 13259384

[B27] Yellon RF Branstetter BF 4th Prospective blinded study of computed tomography in congenital aural atresia Int J Pediatr Otorhinolaryngol 2010 74 1286 1291 10.1016/j.ijporl.2010.08.006 20864187

[B28] Coré N Caubit X Metchat A Boned A Djabali M Fasano L Tshz1 is required for axial skeleton, soft palate and middle ear development in mice Dev Biol 2007 308 407 420 10.1016/j.ydbio.2007.05.038 17586487

[B29] Hill EW Gu J McGivney BA MacHugh DE Targets of selection in the Thoroughbred genome contain exercise-relevant gene SNPs associated with elite racecourse performance Anim Genet 2010 41 56 63 21070277 10.1111/j.1365-2052.2010.02104.x

[B30] Bellone RR Forsyth G Leeb T Archer S Sigurdsson S Imsland F Mauceli E Engensteiner M Bailey E Sandmeyer L Grahn B Lindblad-Toh K Wade CM Fine-mapping and mutation analysis of TRPM1: a candidate gene for leopard complex (LP) spotting and congenital stationary night blindness in horses Brief Funct Genomics 2010 9 193 207 10.1093/bfgp/elq002 20353955

[B31] Tryon RC White SD Bannasch DL Homozygosity mapping approach identifies a missense mutation in equine cyclophilin B (PPIB) associated with HERDA in the American Quarter Horse Genomics 2007 90 93 102 10.1016/j.ygeno.2007.03.009 17498917

[B32] Brooks SA Gabreski N Miller D Brisbin A Brown HE Streeter C Mezey J Cook D Antczak DF Whole-genome SNP association in the horse: identification of a deletion in myosin Va responsible for Lavender Foal Syndrome PLoS Genet 2010 6 e1000909 10.1371/journal.pgen.1000909 20419149 PMC2855325

[B33] Marklund L Moller MJ Sandberg K Andersson L A missense mutation in the gene for melanocyte-stimulating hormone receptor (MC1R) is associated with the chestnut coat color in horses Mamm Genome 1996 7 895 899 10.1007/s003359900264 8995760

[B34] Wagner HJ Reissmann M New polymorphism detected in the horse MC1R gene Anim Genet 2000 31 289 290 10.1046/j.1365-2052.2000.00655.x 11086549

[B35] Brooks SA Bailey E Exon skipping in the KIT gene causes a Sabino spotting pattern in horses Mamm Genome 2005 16 893 902 10.1007/s00335-005-2472-y 16284805

[B36] Brooks SA Lear TL Adelson DL Bailey E A chromosome inversion near the KIT gene and the Tobiano spotting pattern in horses Cytogenet Genome Res 2007 119 225 230 10.1159/000112065 18253033

[B37] Makvandi-Nejad S Hoffman GE Allen JJ Chu E Gu E Chandler AM Loredo AI Bellone RR Mezey JG Brooks SA Sutter NB Four loci explain 83% of size variation in the horse PLoS One 2012 7 e39929 10.1371/journal.pone.0039929 22808074 PMC3394777

[B38] Signer-Hasler H Flury C Haase B Burger D Simianer H Leeb T Rieder S A genome-wide association study reveals loci influencing height and other conformation traits in horses PLoS One 2012 7 e37282 10.1371/journal.pone.0037282 22615965 PMC3353922

[B39] Spirito F Charlesworth A Linder K Ortonne JP Baird J Meneguzzi G Animal models for skin blistering conditions: absence of laminin 5 causes hereditary junctional mechanobullous disease in the Belgian horse J Invest Dermatol 2002 119 684 691 10.1046/j.1523-1747.2002.01852.x 12230513

[B40] Brunberg E Andersson L Cothran G Sandberg K Mikko S Lindgren G A missense mutation in PMEL17 is associated with the Silver coat color in the horse BMC Genet 2006 7 46 17029645 10.1186/1471-2156-7-46 PMC1617113

[B41] Graves KT Henney PJ Ennis RB Partial deletion of the LAMA3 gene is responsible for hereditary junctional epidermolysis bullosa in the American Saddlebred Horse Anim Genet 2009 40 35 41 10.1111/j.1365-2052.2008.01795.x 19016681

[B42] Shin EK Perryman LE Meek K A kinase-negative mutation of DNA-PK(CS) in equine SCID results in defective coding and signal joint formation J Immunol 1997 158 3565 3569 9103416

[B43] Aleman M Riehl J Aldridge BM Lecouteur RA Stott JL Pessah IN Association of a mutation in the ryanodine receptor 1 gene with equine malignant hyperthermia Muscle Nerve 2004 30 356 365 10.1002/mus.20084 15318347

[B44] Gu J MacHugh DE McGivney BA Park SD Katz LM Hill EW Association of sequence variants in CKM (creatine kinase, muscle) and COX4I2 (cytochrome c oxidase, subunit 4, isoform 2) genes with racing performance in Thoroughbred horses Equine Vet J 2010 42 569 75 10.1111/j.2042-3306.2010.00181.x 21059062

[B45] McCue ME Valberg SJ Miller MB Wade C DiMauro S Akman HO Mickelson JR Glycogen synthase (GYS1) mutation causes a novel skeletal muscle glycogenosis Genomics 2008 91 458 466 10.1016/j.ygeno.2008.01.011 18358695 PMC2430182

[B46] Cannon SC Hayward LJ Beech J Brown RH Jr Sodium channel inactivation is impaired in equine hyperkalemic periodic paralysis J Neurophysiol 1995 73 1892 1899 7623088 10.1152/jn.1995.73.5.1892

[B47] Orr N Back W Gu J Leegwater P Govindarajan P Conroy J Ducro B Van Arendonk JA MacHugh DE Ennis S Hill EW Brama PA Genome-wide SNP association-based localization of a dwarfism gene in Friesian dwarf horses Anim Genet 2010 41 2 7 21070269 10.1111/j.1365-2052.2010.02091.x

[B48] Cook D Brooks S Bellone R Bailey E Missense mutation in exon 2 of SLC36A1 responsible for champagne dilution in horses PLoS Genet 2008 4 e1000195 10.1371/journal.pgen.1000195 18802473 PMC2535566

[B49] Hansen M Knorr C Hall AJ Broad TE Brenig B Sequence analysis of the equine SLC26A2 gene locus on chromosome 14q15-->q21 Cytogenet Genome Res 2007 118 55 62 10.1159/000106441 17901700

[B50] Yang GC Croaker D Zhang AL Manglick P Cartmill T Cass D A dinucleotide mutation in the endothelin-B receptor gene is associated with lethal white foal syndrome (LWFS); a horse variant of Hirschsprung disease Hum Mol Gene 1998 7 1047 1052 10.1093/hmg/7.6.1047 9580670

[B51] Hill EW McGivney BA Gu J Whiston R Machugh DE A genome-wide SNP association study confirms a sequence variant (g.66493737C > T) in the equine myostatin (MSTN) gene as the most powerful predictor of optimum racing distance for Thoroughbred racehorses BMC Genomics 2010 11 552 10.1186/1471-2164-11-552 20932346 PMC3091701

[B52] Mariat D Taourit S Guérin G A mutation in the MATP gene causes the cream coat colour in the horse Genet Sel Evol 2003 35 119 133 10.1186/1297-9686-35-1-119 12605854 PMC2732686

[B53] Rieder S Taourit S Mariat D Langlois B Guérin G Mutations in the agouti (ASIP), the extension (MC1R), and the brown (TYRP1) loci and their association to coat color phenotypes in horses (Equus caballus) Mamm Genome 2001 12 450 455 10.1007/s003350020017 11353392

[B54] Andersson LS Larhammar M Memic F Wootz H Schwochow D Rubin CJ Patra K Arnason T Wellbring L Hjälm G Imsland F Petersen JL McCue ME Mickelson JR Cothran G Ahituv N Roepstorff L Mikko S Vallstedt A Lindgren G Andersson L Kullander K Mutations in DMRT3 affect locomotion in horses and spinal circuit function in mice Nature 2012 488 642 646 10.1038/nature11399 22932389 PMC3523687

[B55] Rosengren Pielberg G Golovko A Sundström E Curik I Lennartsson J Seltenhammer MH Druml T Binns M Fitzsimmons C Lindgren G Sandberg K Baumung R Vetterlein M Strömberg S Grabherr M Wade C Lindblad-Toh K Pontén F Heldin CH Sölkner J Andersson L A cis-acting regulatory mutation causes premature hair graying and susceptibility to melanoma in the horse Nat Genet 2008 40 1004 1009 10.1038/ng.185 18641652

[B56] Nielsen R Bustamante C Clark AG Glanowski S Sackton TB Hubisz MJ Fledel-Alon A Tanenbaum DM Civello D White TJ J Sninsky J Adams MD Cargill M A scan for positively selected genes in the genomes of humans and chimpanzees PLoS Biol 2005 3 el70 10.1371/journal.pbio.0030170 PMC1088278 15869325

[B57] Li L Stoeckert CJ Jr Roos DS OrthoMCL: identification of ortholog groups for eukaryotic genomes Genome Res 2003 13 2178 2189 10.1101/gr.1224503 12952885 PMC403725

[B58] Macfadden BJ Fossil Horses: Systematics, Paleobiology, and Evolution of the Family Equidae 1994 Cambridge:Cambridge University Press

[B59] Macfadden BJ Evolution. Fossil horses--evidence for evolution Science 2005 307 1728 1730 10.1126/science.1105458 15774746

[B60] Patel RK Jain M NGS QC Toolkit: A Toolkit for Quality Control of Next Generation Sequencing Data PLoS One 2012 7 e30619 10.1371/journal.pone.0030619 22312429 PMC3270013

[B61] Li H Durbin R Fast and accurate short read alignment with Burrows-Wheeler Transform Bioinformatics 2009 25 1754 1760 10.1093/bioinformatics/btp324 19451168 PMC2705234

[B62] McKenna A Hanna M Banks E Sivachenko A Cibulskis K Kernytsky A Garimella K Altshuler D Gabriel S Daly M DePristo MA The Genome Analysis Toolkit: A MapReduce framework for analyzing next-generation DNA sequencing data Genome Res 2010 20 1297 1303 10.1101/gr.107524.110 20644199 PMC2928508

[B63] Luo R Liu B Xie Y Li Z Huang W Yuan J He G Chen Y Pan Q Liu Y Tang J Wu G Zhang H Shi Y Liu Y Yu C Wang B Lu Y Han C Cheung DW Yiu SM Peng S Xiaoqian Z Liu G Liao X Li Y Yang H Wang J Lam TW Wang J SOAPdenovo2: an empirically improved memory-efficient short-read de novo assembler Gigascience 2012 1 18 10.1186/2047-217X-1-18 23587118 PMC3626529

[B64] Li H Handsaker B Wysoker A Fennell T Ruan J Homer N Marth G Abecasis G Durbin R 1000 Genome Project Data Processing Subgroup The Sequence alignment/map (SAM) format and SAMtools Bioinformatics 2009 25 2078 2079 10.1093/bioinformatics/btp352 19505943 PMC2723002

[B65] Cingolani P Platts A Wang le L Coon M Nguyen T Wang L Land SJ Lu X Ruden DM A program for annotating and predicting the effects of single nucleotide polymorphisms, SnpEff: SNPs in the genome of Drosophila melanogaster strain w^1118^; iso-2; iso-3 Fly(Austin) 2012 6 80 92 22728672 10.4161/fly.19695 PMC3679285

[B66] Purcell S Neale B Todd-Brown K Thomas L Ferreira MA Bender D Maller J Sklar P de Bakker PI Daly MJ Sham PC PLINK: a toolset for whole-genome association and population-based linkage analysis Amer J Hum Genet 2007 81 559 575 10.1086/519795 17701901 PMC1950838

[B67] Stamatakis A RAxML-VI-HPC: maximum likelihood-based phylogenetic analyses with thousands of taxa and mixed models Bioinformatics 2006 22 2688 2690 10.1093/bioinformatics/btl446 16928733

[B68] Stamatakis A Aberer AJ Goll C Smith SA Berger SA Izquierdo-Carrasco F RAxML-Light: a tool for computing terabyte phylogenies Bioinformatics 2012 28 2064 2066 10.1093/bioinformatics/bts309 22628519 PMC3400957

[B69] Tamura K Stecher G Peterson D Filipski A Kumar S MEGA6: Molecular Evolutionary Genetics Analysis Version 6.0 Mol Biol Evol 2013 30 2725 2729 10.1093/molbev/mst197 24132122 PMC3840312

[B70] Ihaka R Gentleman R R: A Language for Data Analysis and Graphics J Comput Graph Stat 1996 5 299 314

[B71] Earl DA Vonholdt BM STRUCTURE HARVESTER: a website and program for visualizing STRUCTURE output and implementing the Evanno method Conserv Genet Resour 2012 4 359 361 10.1007/s12686-011-9548-7

[B72] Rosenberg NA DISTRUCT: a program for the graphical display of population structure Mol Ecol Notes 2004 4 137 138

[B73] Yim HS Cho YS Guang X Kang SG Jeong JY Cha SS Oh HM Lee JH Yang EC Kwon KK Kim YJ Kim TW Kim W Jeon JH Kim SJ Choi DH Jho S Kim HM Ko J Kim H Shin YA Jung HJ Zheng Y Wang Z Chen Y Chen M Jiang A Li E Zhang S Hou H Minke whale genome and aquatic adaptation in cetaceans Nat Genet 2014 46 88 92 24270359 10.1038/ng.2835 PMC4079537

[B74] Ji R Cui P Ding F Geng J Gao H Zhang H Yu J Hu S Meng H Monophyletic origin of domestic bactrian camel (Camelus bactrianus) and its evolutionary relationship with the extant wild camel (Camelus bactrianus ferus) Anim Genet 2009 40 377 382 10.1111/j.1365-2052.2008.01848.x 19292708 PMC2721964

[B75] Yang Z PAML 4: phylogenetic analysis by maximum likelihood Mol Biol Evol 2007 24 1586 1591 10.1093/molbev/msm088 17483113

[B76] Zhang J Nielsen R Yang Z Evaluation of an improved branch-site likelihood method for detecting positive selection at the molecular level Mol Biol Evol 2005 22 2472 2479 10.1093/molbev/msi237 16107592

