# Supplementary material for: Whole genome sequence and analysis of the Marwari horse breed and its genetic origin
Source: BMC Genomics. 2014 Dec 8;15(Suppl 9):S4. doi: 10.1186/1471-2164-15-S9-S4 (PMC4290615; doi:10.1186/1471-2164-15-S9-S4)
Supplement: Additional file 2 [file 1471-2164-15-S9-S4-S2.docx]

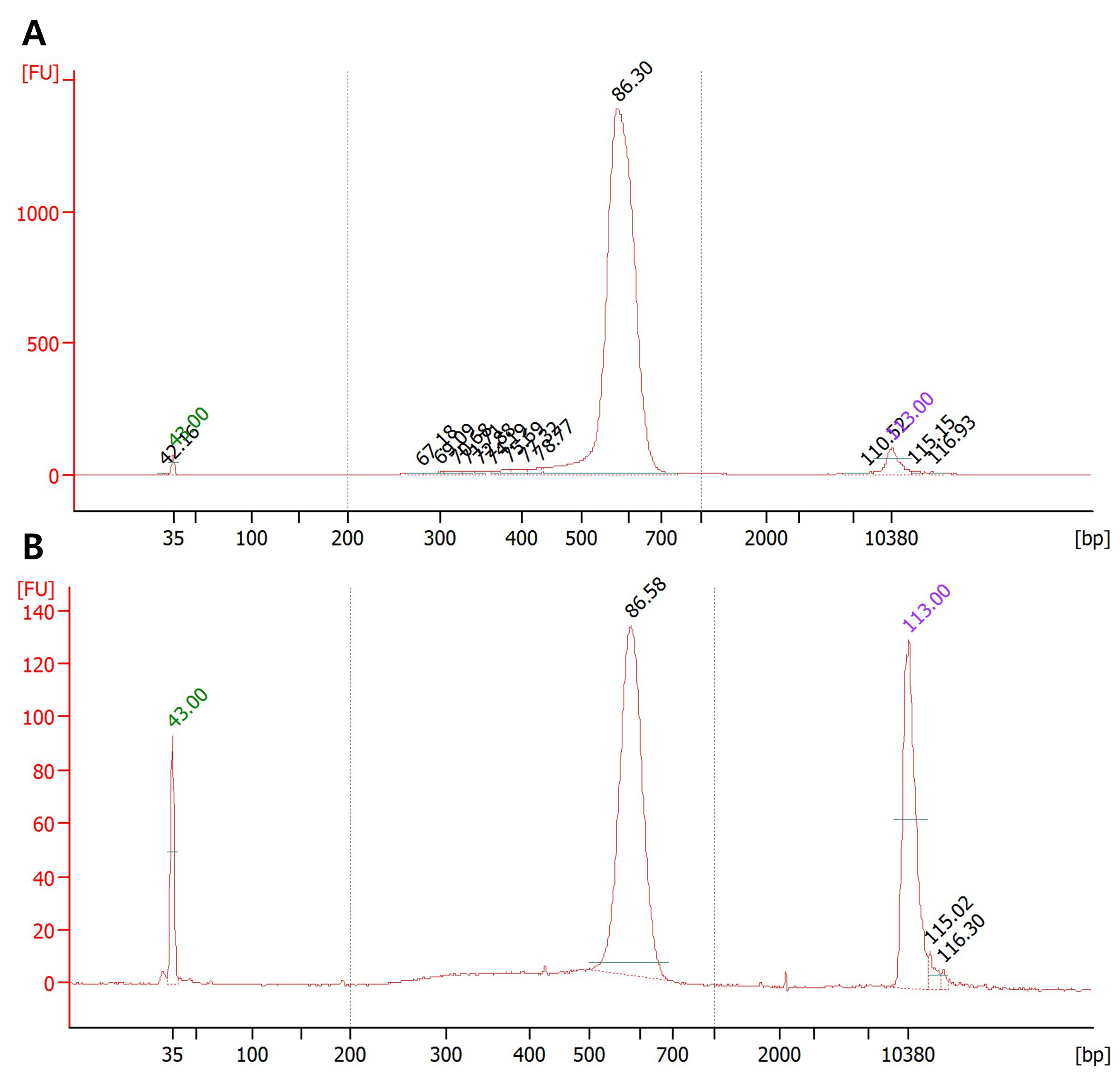


**Supplementary Figure 1. The BioAnalzer profiles of two librarys.** Figures indicating fluorescence unit (FU) and DNA fragment size. Numbers mean the area under the peak within the regions. Peaks of 35bp (green) and 10,380bp (purple) are lower andupper markers. (A) The BioAnalyzer profile of library targeting fragment size of 576 bp (insert size of 456 bp) (B) The BioAnalyzer profile of library targeting fragment size of 582 bp (insert size of 462bp)


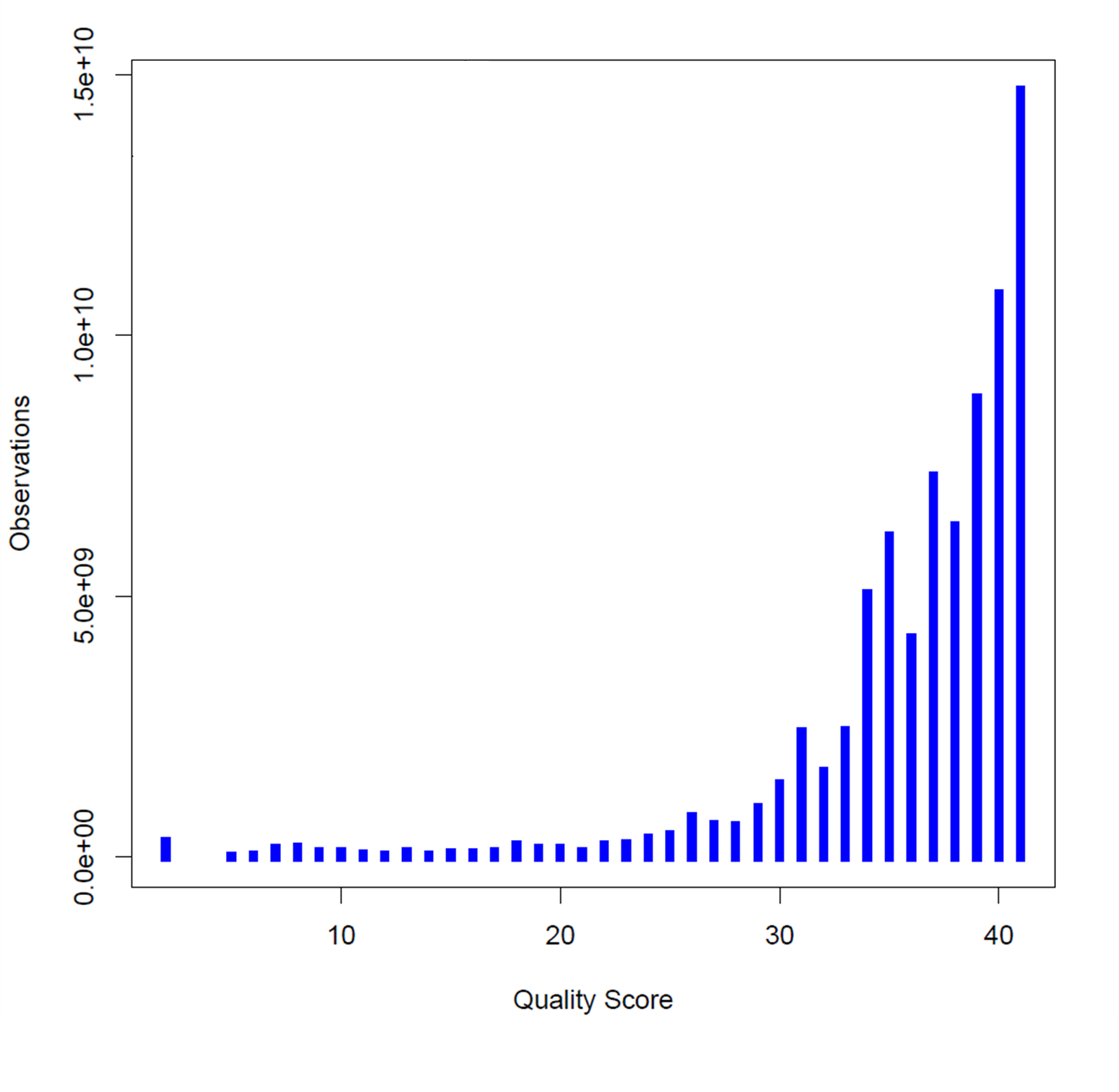


**Supplementary Figure 2. Base quality distribution.** Figure indicating the distribution of phred quality score and number of bases.

**Supplementary Figure 3. Mapping quality distribution of mapped reads.** The mode value of mapping quality is 60 (83.61% of total), and average mapping quality is 53.16.

**Supplementary Figure 4. Mapping rate distribution.** The Mapping rates were dropped when increasing the mapping quality cutoff. We used unfiltered data for next analysis.


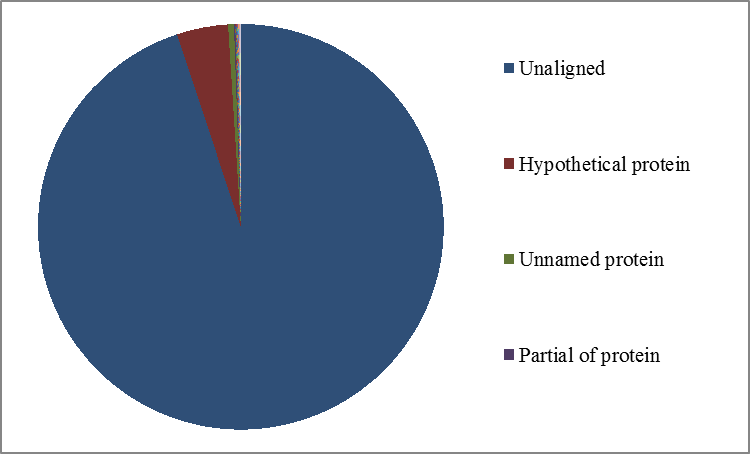


**Supplementary Figure 5. *De novo* assembly of unmapped reads.** A total of 25,614 novel contigs were analyzed using BLAST to a NCBI protein database.


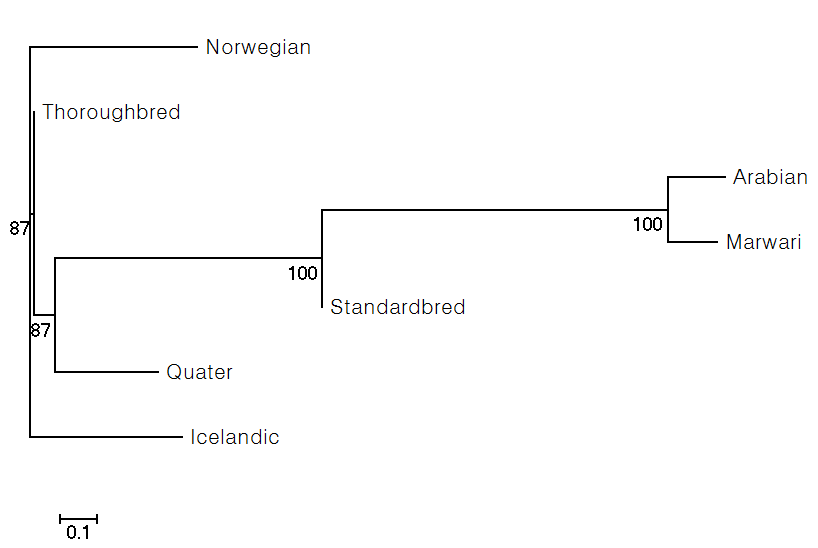


**Supplementary Figure S6. Maximum likelihood tree calculated from 25,854 single nucleotide polymorphisms in whole genome data of seven horses.** Maximum likelihood tree created from 25,854 single nucleotide polymorphisms. Percent bootstrap result supporting all the branches calculated from 100 replicates is shown.
